# Thymoma size significantly affects the survival, metastasis and effectiveness of adjuvant therapies: a population based study

**DOI:** 10.18632/oncotarget.24315

**Published:** 2018-01-23

**Authors:** Dongliang Bian, Feng Zhou, Weiguang Yang, Kaixuan Zhang, Linsong Chen, Gening Jiang, Peng Zhang, Chunyan Wu, Ke Fei, Lei Zhang

**Affiliations:** ^1^ Department of Thoracic Surgery, Shanghai Pulmonary Hospital, Tongji University, Shanghai 200433, P.R. China; ^2^ School of Medicine, Tongji University, Shanghai 200092, P.R. China; ^3^ Department of Pathology, Shanghai Pulmonary Hospital, Tongji University, Shanghai 200433, P.R. China

**Keywords:** thymoma, tumor size, surgery, postoperative radiotherapy, chemotherapy

## Abstract

**Background:**

Thymoma, though a rare tumor disease, is the most common tumor of the anterior mediastinum. However, tumor size, as a critical factor, has been underestimated.

**Results:**

Age, advanced tumor stage, and preoperative radiotherapy were poor prognostic factors of overall survival (OS) and disease specific survival (DSS) (*P* < 0.05 for all). Besides, tumor size was significantly related to survival. The larger tumor size indicated the less OS and DSS (*P* < 0.001 for all). Multivariate analysis revealed elder age, advanced stage, larger size were independent adverse predictors for survival (*P* < 0.05 for all). Logistic analysis revealed larger tumor size had greater rate of metastasis (*P* < 0.001). In the group with tumors smaller than 90mm, chemotherapy was a negative predictive factor of DSS (*P* < 0.05 for all), and it significantly decreased OS especially with tumor sizes between 50 and 90 mm (*P* < 0.001).

**Materials and Methods:**

A total of 1,272 thymoma patients were enrolled from the Surveillance, Epidemiology, and End Results (SEER) database. Survival based on thymoma size and other characteristics of tumors were analyzed by univariate and multivariate analysis. Correlation between thymoma size and thymoma metastatic status was contributed by logistic regression analysis. The efficiency of adjuvant therapy was analysis by stratification analysis.

**Conclusions:**

Thymoma size could predict postoperative survival and guide chemotherapeutic regimens of patients. Larger tumor size indicated worse survival and higher metastatic rate. If thymoma is smaller than 90mm, traditional chemotherapy should be prohibited. While chemotherapy could be performed moderately when thymoma larger than 90 mm.

## INTRODUCTION

Thymoma is a rare disease, characterized by remarkable morphological heterogeneity and unique biological behavior. A study by the United States National Cancer Institute (NCI) and information from the Surveillance, Epidemiology, and End Results (SEER) database has shown that the incidence of thymoma in the United States is 0.15 case per 100,000 persons [1]. However, because thymoma is the most common thymus tumor in the anterior mediastinum [1], it has a crucial status in mediastinal disease.

In many kinds of solid tumors, tumor size is a key factor in predicting patients’ prognosis, and plays an important role in tumors’ clinical classifications [2–5]. However, none of these classifications of thymoma take into consideration the correlation between the thymoma size and the prognosis of patients [6–9]. Presently, surgeons and physicians are not able to consistently predict the prognosis and metastatic status of thymoma patients based on radiographic testing nor are they able to make detailed therapeutic regimens preoperatively. Clearly, establishing additional predictive factors would be extremely beneficial in the identification and treatment of thymoma. The most effective therapy for local and locally advanced thymoma is complete surgical resection [6, 10, 11]. Few studies have focused retrospectively or prospectively on the effectiveness of chemotherapy for thymoma. Thus, the role of chemotherapy for thymoma is still vague, especially the success rate as related to different thymoma sizes [10]. Meanwhile, some previous studies reported that postoperative radiotherapy (PORT) could prevent or postpone postoperative recurrence, and PORT could improve the survival of patients with advanced stages and incomplete resection. However, other studies showed that PORT had a negative effect on survival of patients who received complete resection or in the Masaoka stage I and II [11–14]. Thus, prognostic factors of thymoma and adjuvant treatments effectiveness should be discussed deeply.

In the present study, we attempted to explore the correlation between tumor size, metastatic status and survival outcomes. We also wanted to investigate the effect of chemotherapy on thymomas based on different tumor sizes. These results may assist surgeons and physicians in predicting the patients’ survival and choosing adjuvant treatments via computer tomography (CT) and magnetic resonance imaging (MRI) preoperatively. In addition, we also identified the effectiveness of radiotherapy for thymoma patients in different stages, however, it had no correlation with thymoma size.

## RESULTS

### Patient baseline characteristics

A total of 1,272 patients met the inclusion and exclusion criteria. The patients were divided into 3 tumor-size groups according to 50 and 90 mm 2 cutoff values (0–50 mm, vs 51–90 mm, vs ≥ 91 mm). The baseline and characteristics of the patients are shown in Table 1. There were 630 males and 642 females, with a median age of 56 years (range, 12–90 years). Among all the patients, 157 had distant metastasis beyond the thoracic cavity, and 1,115 patients had none. Metastasis including distant organs or distant lymph nodes invasion was diagnosed by preoperative radiographic testing and surgical findings. There were 696 patients who received radiotherapy and 576 who did not; 37 patients received radiotherapy before surgery, and 659 received PORT. There were 278 patients who received chemotherapy, and 994 who did not. Based on the different invasive status of thymomas, the patients were classified into 3 stages: L, R, and D. The SEER summary stage classification was defined as the same as Masaoka Stage: Masaoka Stage I: L; Masaoka Stage II-III: R; Masaoka Stage IV: D [11]. There were 454 patients in Stage I, 661 in Stage II–III, 157 in Stage IV. According to a chi-square test, the distribution of patients in the 3 size-groups considering these factors had discrepancies. This indicated that the development and advancement of thymoma were indeed related to tumor size. The data is listed in Table 1.

**Table 1:** Patient characteristics in each group divided by tumor size

Tumor Size (mm)		0–50	51–90	≥ 91	Total	*P* value
**Number**		390	575	307	1272	
**Age (years)**						< 0.001
	Median	58	56	52	56	
	Range	18-89	12-90	14-86	12-90	
**Gender**						0.028
	Male	178	281	171	630	
	Female	212	294	136	642	
**Masaoka Stage**						< 0.001
	I	183	190	81	454	
	II-III	182	317	162	661	
	IV	25	68	64	157	
**WHO Grade**						0.191
	A	35	48	21	104	
	AB	69	94	54	217	
	B1	43	82	47	172	
	B2	72	83	41	196	
	B3	62	117	47	357	
	Unknown	109	151	97	307	
**Chemotherapy**						< 0.001
	Without	349	444	201	994	
	With	41	131	106	278	
**Radiotherapy**						0.002
	Without	201	243	132	576	
	Preoperative	5	16	16	37	
	Postoperative	184	316	159	659	

*P* value was calculated by chi-square test.

### Survival analysis

**Figure 1:**
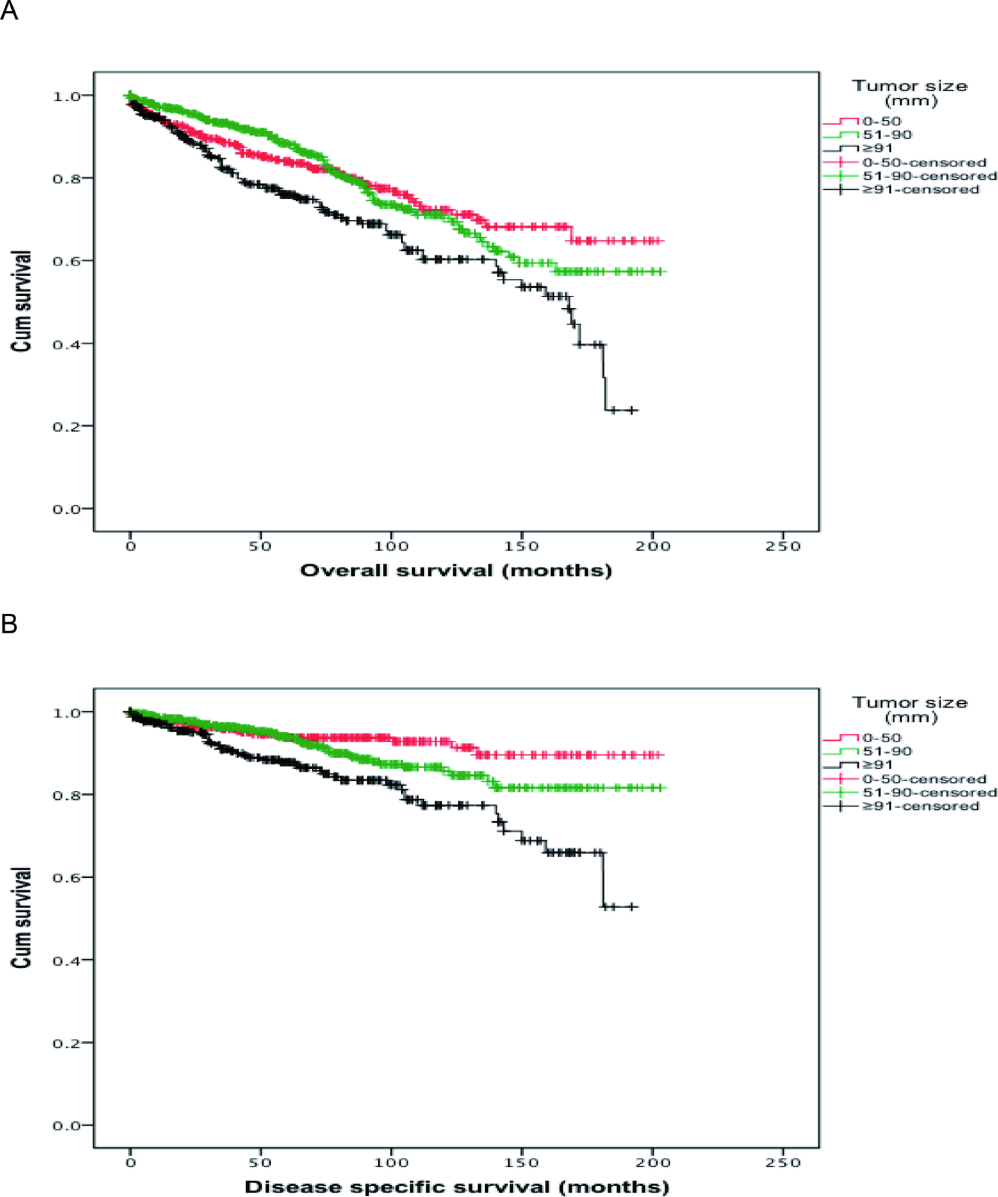
(**A**) Overall survival (OS) curves of patients according to tumor size. The red curve represents the overall survival of patients whose thymoma size smaller than 50mm; the green curve represents the overall survival of patients whose thymoma size between 51mm and 90mm; the black curve represents the overall survival of patients whose thymoma size larger than 91mm. (**B**) Disease specific survival (DSS) curves of patients according to tumor size. The red curve represents the disease specific survival of patients whose thymoma size smaller than 50mm; the green curve represents the disease specific survival of patients whose thymoma size between 51mm and 90mm; the black curve represents the disease specific survival of patients whose thymoma size larger than 91mm.

To evaluate the factors affecting the survival of thymoma patients, OS and DSS were analyzed (see Table 2). Based on the univariate analysis, patients’ gender and WHO classification had no significant correlation with the OS and DSS. Patients’ age, Masaoka Stage and size of thymoma, and adjuvant therapies were significantly associated with the OS and DSS. With increasing age, upgrading tumor stage, larger tumor size and occurrence of distant metastasis (Stage IV), both OS (*P* < 0.001, for all) and DSS (age, *P* = 0.015; *P* < 0.001, for others) were decreased significantly. As shown by the survival curves in Figure 1 (made by Kaplan-Meier method), OS and DSS of patients decreased significantly (*P* < 0.001, respectively) with larger thymoma size. In addition, compared with patients without chemotherapy, the patients who received chemotherapy had lower OS (153.1 vs 131.9 months, *P* = 0.005). The same trend was observed in DSS (184.3 vs 151.4 months, *P* < 0.001). Patients with preoperative radiotherapy had lower OS and DSS than patients with PORT or without radiotherapy (*P* = 0.007 and *P* < 0.001, respectively).

**Table 2:** Univariate and Multivariate analysis for OS and DSS of thymoma patients

			Univariate analysis			Multivariate analysis		
			Survival Rate (%)	Mean Time (Month)	*P* value	HR	95% CI	*P* value
**OS**	**Age**				**< 0.001**			
		< 60	83.8	162.5		1.000		
		≥ 60	71.9	124.8		2.301	1.806-2.930	**< 0.001**
	**Gender**				0.390			
		Male	79.4	151.6				
		Female	78.2	144.3				
	**Masaoka Stage**				**< 0.001**			
	**+**	I	90.3	175.5		1.000		
		II-III	74.3	142.0		2.632	1.870–3.705	**< 0.001**
		IV	64.3	114.0		3.870	2.507–5.974	**< 0.001**
	**WHO Grade**				0.244			
		A	76.0	120.5				
		AB	86.2	155.9				
		B1	79.1	127.3				
		B2	85.2	136.7				
		B3	77.0	139.8				
	**Size (mm)**				**< 0.001**			
		0-50	81.7	156.5		1.000		
		51–90	81.0	153.6		1.006	0.671–1.224	0.521
		> 90	70.4	128.0		1.317	1.056–1.813	0.042
	**Chemo**				0.005			
		Without	80.6	153.1		1.000		
		With	72.3	131.9		1.364	1.031–1.805	0.030
	**Radio**				0.007			
		Without	80.4	147.5		1.000		
		Postoperative	78.5	151.9		1.301	0.746–2.267	0.353
		Preoperative	59.5	110.1		1.479	1.127–1.873	0.003
**DSS**	**Age**				0.015	1.000		
		< 60	91.7	180.8				
		≥ 60	90.0	167.0		1.583	1.095–2.289	0.015
	**Gender**				0.669			
		Male	91.1	177.2				
		Femle	90.8	175.1				
	**Masaoka Stage**				**< 0.001**			
		I	97.6	196.5		1.000		
		II-III	89.7	175.4		3.799	1.983–7.277	**< 0.001**
		IV	77.1	134.9		7.119	3.429–14.782	**< 0.001**
	**WHO Grade**				0.181			
		A	93.3	150.6				
		AB	96.3	186.7				
		B1	89.5	143.6				
		B2	93.4	155.0				
		B3	89.8	166.5				
	**Size (mm)**				**< 0.001**			
		0–50	94.4	187.6		1.000		
		51–90	91.8	180.0		1.212	1.007–2.023	0.027
		> 90	85.0	154.7		1.742	1.024–2.966	0.041
	**Chemo**				**< 0.001**			
		Without	93.5	184.3		1.000		
		With	82.0	151.4		2.399	1.618–3.558	**< 0.001**
	**Radio**							
		Without	92.2	177.9	**< 0.001**	1.000		
		Postoperative	90.0	178.3		0.709	0.479–1.051	0.087
		Preoperative	73.0	134.0		1.675	1.335–3.393	0.027

OS, overall survival; DSS, disease-specific survival; HR, hazard ratio; CI, confidence interval; Chemo: chemotherapy; Radio: radiotherapy.

The variables which significantly affected survival in univariate analysis were calculated by multivariate analysis. Based on the Cox regression analysis, the hazard ratio (HR) of increased thymoma size for OS showed a significant difference, especially tumor size larger than 90 mm (HR: 1.317; 95% CI: 1.056–1.813; *P* = 0.042). It was concluded that the thymoma size was an independent prognostic factor for OS. With the larger thymoma size, especially when the tumor size was greater than 90 mm, patients had a worse prognosis with OS. Apart from this, HR of greater age of patients (< 60 vs ≥ 60 years) for OS showed a significant difference between these 2 age groups (HR: 2.301; 95% CI: 1.806–2.930; *P* < 0.001). HR of upgrading stages for OS showed significant discrepancies among the 3 stage-groups (HR: 2.632, 95% CI: 1.870–3.705, *P* < 0.001; HR: 3.870, 95% CI: 2.507–5.974, *P* < 0.001, respectively). HR of OS for different radiotherapy regimens (without vs with preoperative radiotherapy, HR: 1.479; 95% CI: 1.127–1.873; *P* = 0.003) and different chemotherapy regimens (without vs with chemotherapy, HR: 1.364; 95% CI: 1.031–1.805; *P* = 0.030) also exhibited significant discrepancies. These indicated that the age of the patient, the stage and size of the thymoma, and performing adjuvant chemotherapy or neoadjuvant radiotherapy were independent prognostic factors for OS. In addition, larger tumor size, elder patients’ diagnostic age, tumor stage upgrading, and performing chemotherapy or preoperative radiotherapy may shorten thymoma patients’ OS time and decrease OS rate.

HR of DSS for larger tumor size showed a significant difference among the 3 size-groups (0–50 mm, vs 51–90 mm, vs ≥ 91 mm) (HR: 1.212, 95% CI: 1.007–2.023, *P* = 0.027, HR: 1.742, 95% CI: 1.024–2.966, *P* = 0.041, respectively). This result indicated that tumor size was an independent prognostic factor for DSS. With the larger tumor size, patients had worse DSS. Besides, HR of DSS for increasing age (< 60 vs ≥ 60 years) showed a significant difference (HR: 1.583; 95% CI: 1.095–2.289; *P* = 0.015). There was also a significant difference in HR of DSS for upgrading Masaoka Stage (I, II–III, IV) (HR: 3.799, 95% CI: 1.983–7.277, *P* < 0.001; HR: 7.119; 95% CI: 3.429–14.782, *P* < 0.001, respectively). HR of DSS for performing chemotherapy (without vs with, HR: 2.399; 95% CI: 1.618–3.558; *P* < 0.001) or preoperative radiotherapy (without vs with, HR: 1.675; 95% CI: 1.335–3.393; *P* = 0.027) showed a significant difference as well. These results indicated that the size and stage of tumor, diagnostic age of patients, and performing chemotherapy or preoperative radiotherapy were independent prognostic factors for DSS. These variables lead to poor patients’ DSS indeed.

### Correlation of tumor size and metastatic status

Logistic regression model was performed to analyze the correlation between the thymoma size and metastatic status of the patients. “Metastasis” was defined as distant metastasis, including lymph nodes and structures involvement beyond the thoracic cavity. If thymoma invaded mediastinal or thoracic lymph nodes or structures, it would not define as metastasis in the present study. As shown in Table 3, the possibility of metastasis was significantly increased with the increasing thymoma size among the 3 size-groups (OR: 1.958 and 3.845, 95% CI: 1.214–3.157 and 2.356–6.276, *P* < 0.001, respectively). This result indicated that, patients with a tumor smaller than 50 mm experienced metastasis at a rate lower than did patients with tumors larger than 50 mm and 90 mm (an increase of 1.958 and 3.845 times, respectively). The metastatic status and thymoma size had a definite relationship. In cases with indolent tumors, the rate of thymoma metastasis beyond the thoracic cavity is extremely rare. However, the treatments for metastatic patients, palliative or conservative regimens, was significantly different from local and locally advanced thymomas [10]. Surgeons and physicians should perform preoperative thorough check-ups for thymoma patients if thymoma size is larger than 50 mm, especially as large as 90 mm.

**Table 3:** The correlation analysis between tumor size and thymoma metastasis status by Logistic regression

	OR	95% CI	*P* value
**Tumor Size [0–50 (mm)]**	1.000		
**51–90 (mm)**	1.958	1.214-3.157	< 0.001
**> 91 (mm)**	3.845	2.356-6.276	< 0.001

OS, overall survival; DSS, disease-specific survival; OR, odds ratio; CI, confidence interval.

### Tumor size stratification analysis concerning adjuvant therapies (Chemotherapy and PORT)

In addition to size and other characteristics of tumors described above, therapeutic regimens also correlated with survival of thymoma. As we all know, surgical treatment is the most important approach to treating thymoma [6, 10–12]. Since the survival of patients with preoperative radiotherapy was significantly poor, we removed 37 patients who received preoperative radiotherapy from the cohort and re-analyzed the effectiveness of adjuvant therapies within the remaining 1,235 patients. As shown in Table 2, surgically treated thymoma patients who received chemotherapy had worse OS and DSS than the patients who did not receive chemotherapy. Furthermore, in each size-group divided by tumor diameter (50 and 90 mm), the OS and DSS of the patients with or without chemotherapy demonstrated a difference (listed in Table 4). With tumor sizes between 51 mm and 90 mm, patients without adjuvant chemotherapeutic treatment had significantly better OS than the others (157.8 vs 134.1 months, *P* = 0.016). If tumor size is smaller than 50 mm or between 51 mm and 90 mm, patients without adjuvant chemotherapy had significantly better DSS than the remaining patients (0–50 mm: 189.9 vs 163.1 months, *P =* 0.0014; 51–90 mm: 185.4 vs 160.0 months, *P* = 0.007, respectively). However, OS and DSS had no significant difference between patients with and without chemotherapy if their tumor size was larger than 90 mm. These results indicated that chemotherapy had negative effectiveness in treating surgically treated thymoma patients whose tumor size was smaller than 90 mm. These findings indicate that tumor size could act as a predictive factor to guide chemotherapy preoperatively.

**Table 4:** Stratification analysis for OS and DSS based on adjuvant therapies

	Adjuvant Therapy	Number	OS			DSS		
			Rate (%)	Time (Month)	*P* value	Rate (%)	Time (Month)	*P* value
**Size (mm)**	**Chemo**							
**0–50**					0.634			0.014
	Without	348	80.8	153.6		95.7	190.0	
	With	37	73.5	134.9		83.8	163.1	
**51–90**					0.016			0.007
	Without	441	81.9	157.8		93.9	185.4	
	With	118	73.0	134.1		86.4	160.0	
**≥ 91**					0.764			0.067
	Without	197	71.6	132.0		88.3	164.1	
	With	94	72.3	125.8		80.9	139.4	
**Masaoka Stage**	**Radio**							
**I**					0.942			0.188
	Without	294	91.5	176.7		98.6	199.3	
	With	155	89.0	171.5		96.1	187.4	
**II–III**					0.015			0.098
	Without	229	72.1	129.7		88.6	164.4	
	With	409	75.8	147.6		90.7	178.2	
**IV**					0.015			0.049
	Without	53	54.7	96.6		71.7	123.1	
	With	95	69.2	129.8		83.2	147.2	

OS, overall survival; DSS, disease-specific survival; Chemo: chemotherapy; Radio: radiotherapy.

In contrast, in each size-group, there was no difference of OS and DSS found between patients with and without radiotherapy. On the other hand, significant discrepancies of OS and DSS between patients with and without PORT were observed in stage-groups divided by Masaoka Stage. In thymoma in Masaoka Stages II–IV, surgically treated patients with PORT had significantly better OS than those without PORT (*P* = 0.015). However, PORT was ineffective in treating Stage I patients. Detailed data is shown in Table 4.

## DISCUSSION

It is well known that thymoma patients would have improved likelihood of survival if they accepted appropriate treatments, even though the disease has relapsed or metastasized after initial treatments [6, 11]. Therefore, optimizing the treatment strategies based on different characteristics of the thymomas is a reasonable approach to improve the survival rate and prolong the survival time [12]. Surgery is the most important treatment for thymomas [6, 10–12]. The surgical treatment, especially completeness of the surgical resection, could prolong survival time of thymoma patients significantly [6, 10].

As a large population based database, SEER has its advantages to retrospective analysis the lower incident diseases. The SEER program is widely used to calculate the survival of patients based on different characteristics of patients and tumors, such as pulmonary carcinoma, esophageal carcinoma, and gastrointestinal carcinoma etc.. SEER is also a reliable source of information concerning effectiveness of treatments.

The principal purpose of the present study was to evaluate the relation between thymoma size and survival and metastatic rate of patients. Since the thymoma is an indolent tumor, the status of thymoma size has been underestimated. Few studies have researched thymoma size, especially the correlation between thymoma size and the postoperative survival time of the patients. The lack of a standard classification on thymoma size has hindered meaningful comparisons. Keshavjee found that patients with tumors larger than 70 mm had worse prognoses than did patients with smaller tumors [13]. Wu found that tumor size larger than 80 mm is a negative factor upon prognosis [14]. The study by Fukui suggested that tumors larger than 40mm showed significantly worse outcomes in survival [15]. The different cutoff values of thymoma sizes were used in other reports [16–19]. In our research, we found that tumor size is an independent predictive factor for OS and DSS. What is more, the patients with tumor size smaller than 50mm had obviously better OS and DSS, and patients with tumor size larger than 90mm had worse outcomes (*P* < 0.001, respectively). The study by Hwang suggested that tumor size larger than 60mm predicted node metastasis [17]. Furthermore, we observed that according to the two cutoff values of OS, the rate of metastasis and recurrence of thymomas were increased with the greater size of thymomas (OR: 1.958, *P* < 0.001, OR: 3.845, *P* < 0.001). Thus, if the tumor size is larger than 50 mm, especially 90 mm, the metastatic rate of thymomas increases and OS and DSS of the patients decreases significantly. Thymoma is an indolent tumor which rarely metastasizes beyond the thoracic cavity [10]. However, this study suggests that increased thymoma size influences distant metastatic rate. Surgeons and physicians should be particularly attentive to the likelihood of metastasis occurring when thymoma size is larger than 50 mm, especially 90 mm. Tumor size of thymoma, as with any other solid tumor [2–5], is a critical factor in describing the characteristics of the tumor, in predicting prognosis, and in making treatment regimens preoperatively. In addition, according to the significant role of thymoma size, we suggest that the thymoma classification should take into consideration tumor size. Aside from this, with tumors larger than 50 mm, especially 90 mm, the diagnosis of thymomas should take precautions against metastasis. According to these results, we recommend that the status of thymoma size should be considered.

Apart from the tumor size, we also analyzed age and gender as prognostic factors. Previous studies indicated that age and gender were prognostic factors for survival time [20–23]. In this study, we confirmed that patients younger than 60 years had a better prognosis (*P* < 0.001). Multivariate analysis demonstrated that age was an independent predictive factor for thymoma patients’ OS (HR: 2.301; *P* < 0.001), and DSS (HR: 1.583; *P* = 0.015). However, gender had no correlation with OS and DSS.

The therapeutic regimens also correlated with survival of thymoma. Another aspect of our study was to analyze treatment regimens. Some studies indicate that adapted adjuvant treatment regimens could improve survival rate and prolong survival time [7–11]. Findings indicate that adjuvant treatments have intensive correlations with prognosis. Radiotherapy (RT) is important in the postoperative treatment strategy of thymomas. However, which patients respond better to RT is still unclear [10–14]. In our study, we concluded that preoperative radiotherapy can predict a significant negative prognosis for OS and DSS. According to PORT, some researchers believe that complete resection alone is a sufficient treatment for earlier stage tumors (Masaoka Stage I or II), and resected lesion with positive margins would be performed PORT [13, 24]. Thymomas in Masaoka Stage III or IV should also receive PORT no matter the resected margins [14]. Some researchers have concluded that thymoma patients do not need to get further treatment including PORT [12, 25–27], citing the fact that PORT seems to have little advantage postoperatively [12, 16, 28, 29]. Unfortunately, the analysis of thymomas based on the SEER database could not evaluate the status of pathological resected margins. In our research, we concluded that different stages of thymomas being given different adjuvant treatments had significantly different outcomes. The OS of thymoma patients in Masaoka Stages II-IV with PORT was better (*P* = 0.015, for all). In addition, the DSS of patients in Masaoka Stage IV with PORT had significant advantages (*P* = 0.049). Based on our analysis, we recommend the patients in Masaoka Stages II-IV should undergo PORT, which would promote the chance of survival significantly. Moreover, we found that the effectiveness of PORT was determined by tumor stage, rather than tumor size.

Previous studies concerning treating thymomas with adjuvant chemotherapy are controversial [9, 10, 16]. Some small population studies showed that the response to traditional chemotherapy was not acceptable [10, 30]. However, we found that according to tumor size, chemotherapy predicted different prognosis of OS. Especially patients with tumor size from 51 mm to 90 mm who received adjuvant chemotherapy had significantly poorer OS (157.8 vs 134.1 months, *P* = 0.016). However, when tumor size was smaller than 50 mm or larger than 90 mm, patients who received adjuvant chemotherapy had no significant difference of the OS compared with patients who received no chemotherapy. In cases where tumor size was smaller than 50 mm, patients who received chemotherapy combined with surgery had significantly poorer DSS (190.0 vs 163.1 months, *P* = 0.014), compared with cases where tumor size was from 51 mm to 90 mm, in which chemotherapy predicted poorer DSS (185.4 vs 160.0 months, *P* = 0.007).

Some previous studies reported that induction chemotherapy for locally advanced thymoma could improve the rate of complete resection, decrease the rate of recurrence, and prolong the recurrence-free survival (RFS), OS, and DSS [10, 30–32]. Combined with the results of the present study, we recommend that chemotherapy could be performed to specific locally advanced patients whose tumor size is larger than 90 mm. If the tumor size of patients is smaller than 90 mm, chemotherapy could actually decrease their OS and DSS significantly. This seems to indicate that neoadjuvant chemotherapy should not be prescribed for patients whose tumor size is smaller than 90 mm. The thymoma size had a significant relationship which could assist doctors in choosing reasonable therapeutic regimens preoperatively. However, since the SEER database did not include data on the sequences of chemotherapy and surgery, the effectiveness of neoadjuvant chemotherapy could not be concluded. The assumption of neoadjuvant chemotherapy will be concretely confirmed by the data from our single-center in the next step.

Besides tumor size, tumor stage, recurrence status and adjuvant therapies, Hiroyuki tried to determine the relation of postoperative survival and approaches of surgery for thymoma patients. He found no significant difference of OS, RFS, and recurrence rate for thymoma patients who received video-assisted thoracoscopic surgery (VATS) and those who underwent sternotomy. However, compared with sternotomy, VATS significantly increased the recurrence rate of thymoma in patients with tumors larger than 50 mm [33]. A reasonable explanation seems to be that thymoma size larger than 50 mm could significantly decrease patients’ OS and DSS.

### Limitation

Although it is a large population based database useful for retrospective studies, SEER’s lack of some information, such as scope and margins of surgery, dosage, regimens, side-effects and sequences of adjuvant treatment, and patients’ RFS, limits its applications. For this reason, our analysis did not consider as prognostic tools these factors not supplied by SEER. Nevertheless, the results of correlations between tumor size and patients’ survival are reliable. The problems were caused by these limitations which would be done by utilizing the database from our own single-center in the future studies.

## MATERIALS AND METHODS

We acquired the data for analysis from the SEER program, which is composed of geographically defined, population-based, and central cancer registries in the United States and is operated by local nonprofit organizations under contract with the NCI [1].

The patients included in this analysis were diagnosed with tumors between 1973 and 2014, were without tumor history, and suffered from thymomas as their primary thymus tumor. The inclusion criteria were: (1) patients who suffered from thymomas, (2) thymomas were defined as type A to type B3 according to WHO Classification, (3) the information of the patients, including baseline information, characteristics of tumor, regimens of therapies, follow-up information, etc., was recorded in detail, and (4) patients received surgical treatment. Patients were excluded if: (1) they had a tumor history, (2) the age of patients was younger than 12 years old, (3) the follow-up information of patients was incomplete, and (4) thymomas were defined as carcinoma according to WHO Classification. The parameters of the patients in the cohort obtained from the database included: 1) the year of diagnosis, 2) the age of the patient at diagnosis, 3) gender, 4) WHO grade, 5) SEER summary stage, 6) tumor size (the longest diameter of thymoma), 7) metastatic status at the time of diagnosis, 8) approaches of treatment, 9) whether or not the patient received radiotherapy, especially PORT, 10) whether or not the patient received chemotherapy, 11) time from diagnosis to last contact, and 12) cause of death. The prognostic factors such as TNM stage, margin status of resection, dose and side-effect of radiotherapy and chemotherapy were not included in the SEER database. Therefore, these factors were not analyzed in this research.

Statistical analysis was performed using SPSS software, version 23.0 (SPSS Inc., Chicago, USA). Kaplan-Meier method was used to estimate the overall survival (OS) and disease-specific survival (DSS) of patients. Univariate analysis was performed by log-rank test to predict relationships between parameters and OS or DSS. Cox proportional hazards regression method was used to identify predictors of OS and DSS. Logistic regression model was performed to evaluate correlation between tumor size and metastatic status of thymomas. The time of OS was measured from the date of diagnosis to death from any cause. Patients who were alive at last contact were censored at that date. A two-tailed *P* value < 0.05 was considered statistically significant. The hazard ratio (HR) and odds ratio (OR) were presented with their 95% confidence interval (CI).

## CONCLUSIONS

According to different ages, tumor sizes, thymoma stages, and adjuvant therapies, postoperative thymoma patients manifest broad ranges of survival and metastasis. thymoma size could be considered as an independent prognostic factor. Larger tumor size indicated significantly worse prognoses and high metastatic rate. Thymoma size also affects the efficiency of chemotherapy. Chemotherapy could be performed moderately to prolong patients’ survival in the selected patients whose tumor size larger than 90mm. While it should be inhibited if thymoma size smaller than 90 mm. The efficiency of PORT has no correlation with thymoma size, while, significant OS and DSS advantages are associated with PORT for Masaoka Stage II–IV patients.
